# Efficiency of the original flavonoids composition based on Curcumin in chronic alcohol intoxication

**DOI:** 10.1192/j.eurpsy.2022.2137

**Published:** 2022-09-01

**Authors:** E. Markova, I. Goldina, I. Savkin, M. Knyazheva, A. Smyk

**Affiliations:** Federal State Research Institute of Fundamental and Clinical Immunology Russian Academy of Sciences, Neuroimmunology Lab., Novosibirsk, Russian Federation

**Keywords:** flavonoids, alcoholism

## Abstract

**Introduction:**

Multiple tissue and organ damage induced by ethanol toxic effects requires prescription of a wide range of drugs that have a positive symptomatic effect, while simultaneously increasing the toxic load on the body, which necessitates the search for new approaches to the therapy of alcoholism, possibly with the use of parapharmaceuticals.

**Objectives:**

The anti-inflammatory, antioxidant and neuroprotective properties of curcuminoids, as well as their ability to influence the epigenetic mechanisms of gene expression regulation, can provide a positive therapeutic effect in alcoholism by influencing the pathogenetic mechanisms of this pathology, which determines the relevance and prospects of studying their effects during chronic ethanol intoxication.

**Methods:**

(CBAxC57Bl/6) F1 male mice with 6-month 10% ethanol exposure were undergoing administration of original composition of flavonoids based on Curcumin for 40 days. Animal’s alcohol consumption, behavior and immune parameters were estimated.

**Results:**

As a result of flavonoids original composition administration, correction of the changes in the nervous system functional activity, caused by the chronic toxic effect of ethanol has been achieved (decrease in alcoholic motivation, exploratory behavior stimulation, modulation of the cytokines level in brain, indicating a neuroinflammation decrease). We also registered the immune system functional activity modulation (stimulation of immune response and lymphocytes proliferative activity) in mice with alcohol dependence.

**Conclusions:**

The original composition of flavonoids against the background of long-term alcohol consumption has a positive psychoneuroimmunomodulatory effect, which serves as an experimental substantiation of the prospects of using the composition as an adjuvant in the treatment of chronic alcoholism.

**Disclosure:**

No significant relationships.

